# A deep learning based method for intelligent detection of seafarers' mental health condition

**DOI:** 10.1038/s41598-022-11207-7

**Published:** 2022-05-12

**Authors:** Zhu Zhen, Renda Wang, Wei Zhu

**Affiliations:** 1 Laboratory of Marine Simulation and Control, Dalian Maritime University, Dalian, Liaoning Province China; 2 Navigation College of Dalian Maritime University, Dalian, Liaoning Province China

**Keywords:** Machine learning, Human behaviour

## Abstract

Mental health monitoring of seafarers is an important part of achieving normal development of the ocean shipping industry. In this paper, a dual subjective–objective testing scheme is proposed to achieve a more effective and intelligent assessment of seafarers' mental health status. Firstly, a new seafarers' mental health test scale (SMHT) is revised based on fuzzy factor analysis and the test data of 283 marine practitioners are analyzed using SPSS v24 software; secondly, this paper proposes an intelligent framework module for immersive subjective emotion extraction based on natural language processing, namely semantic summary extraction (SSE), speech emotion extraction (SEE), using hybrid scoring mechanism to obtain semantic and emotion matching values and assist the seafarer mental health scale to obtain the final correction score. The results showed that the assessment results of the SMHT scale exhibited good reliability (Cronbach's alpha of 0.852 $\in (0.80{-}0.90)$ and retest reliability R of $0.873\in (0.85{-}0.90)$) and scale association validity (for SCL-90, ($\text{r}= 0.468{-}0.841)>0.45$). In addition, the calibration rate of the subject-object dual test method was improved by approximately 12.05% compared to the traditional mental health scale. Finally, the advantages and disadvantages of this solution were compared with mental health testing techniques such as CAT, machine learning, SCL-90, and fMRI, and the method demonstrated more accurate psychological testing results, providing a simple and intelligent solution for standardized psychological testing of seafarers.

## Introduction

The slogan of World Mental Health Day 2021 is "Mental Health for All: let's make it a reality". According to IMO, more than 85% of maritime accidents are related to human factors, with fatigue and stress being the key factors contributing to human error^1^. At the same time, mental disorders (depression, anxiety, stress, and post-traumatic stress disorder) are common among seafarers, but the exact extent of their impact is not yet known. The incidence of mental health abnormalities among seafarers is as high as 49.9%^2^, much higher than the average for the normal population. Therefore, effective monitoring of the psychological changes of seafarers and further proposing a solution is an important part of achieving normal development in the ocean. In recent years, with the continuous development of the maritime industry, research on the monitoring of seafarers' mental health has gradually increased, and it is very meaningful to focus on how to effectively detect and assess the mental health literacy of seafarers. Commonly used mental health testing techniques include expert interviews and traditional mental health testing scales. Carotenuto^3^ et al. introduced the Psychological General Well-Being Index PGWBI index, which modified the "non-specific" questionnaire to assess a "specific "non-specific" group of seafarers to evaluate the stress profile of seafarers in the merchant marine; Gibbons^4^ explored the application of computerized adaptive diagnostic screening and computerized adaptive testing in the presence and severity of mental health disorders such as depression, anxiety, and mania. Arkaprabha Sau^5^ used the Python programming language to evaluate and compare five machine learning classifiers, CatBoost, Logistic Regression, Parsimonious Bayes, Random Forest, and Support Vector Machine, and apply them to group mental health detection; Yingchao Shi^6^ based on the relationship between failure mode networks (DMN) and human health conditions, analyzed functional magnetic resonance imaging data of seafarers, proposed an early warning method for human mental subhealth using a dual support vector machine (SVM) model. Liu et al.^7^ proposed a method to assess the psychological status of seafarers using a combination of independent component (IC) fingerprint and support vector machine (SVM). The above research methods generally exhibit the disadvantages of being restricted to a specific group and having insignificant assessment characteristics.

The traditional mental health scale is still an important component of mental health testing methods, but this method can only be analyzed and summarized based on the data characteristics of a specific time and a specific group of interviewees (non-seafarers) and reflects the overall mental health level through a small number of samples, which is not universal and does not complete the transferability of mental health characteristics^8^. Therefore, this paper proposes an intelligent framework for immersive subjective emotion extraction based on natural language processing, and adopts a more suitable dual subjective and objective detection method to replace the traditional mental health scale detection, while providing an intelligent detection scheme for the mental health detection of a specific seafarer population.

## Methods

### Ethics statement

This study was a questionnaire-based observational survey experiment conducted 1 year after the outbreak of COVID-19 at the end of 2019. The study has been approved by the ethics committee of the Navigation College of Dalian Maritime University, all data generated or analyzed during this study are included in this article. All participants were informed of the purpose of the study and were assured that their privacy would be protected prior to the start of the study. Informed consent was obtained from all individual participants included in the study. All methods in this study including experimental procedures, data collection and data processing were carried out in accordance with guidelines and regulations."

### Causes

Despite the rapid progress of modern navigation technology, maritime shipping is still defined as a demanding, stressful and risky type of work, and this occupational group is characterized by long-term sea voyages, irregular biological rhythms, fixed interactions with people, and high levels of work stress, which can have a potential negative impact on the psychological health of seafarers^9^.

The factors influencing the mental health of seafarers are complex and diverse, and this paper provides a preliminary summary of the current factors affecting the life and work status of seafarers through literature analysis^10^ and the semi-interview method^11^. By searching "Mental health of seafarers", "Mental Health Scale", "Mental Health" and "Mental Health" in the web of science and google scholar databases, the paper is a preliminary summary of the factors affecting the current life and work of seafarers. The initial screening of 268 papers matched the theme of "Mental Health", and further screening of the themes of scale assessment and scale creation resulted in 89 relevant papers. An interdisciplinary research approach including sociological research was taken. The main general predisposing factors involved that may be considered inherent to the maritime profession include isolation and loneliness^12^, insufficient shore leave^13^, bullying^14^, fear of unemployment^15^ and separation from family^16^.

Most traditional scales are generally cross-sectional, i.e., prevalence surveys, which do not identify long-term associations between scale characteristics and mental health outcomes, but rather objectively respond to associations collected at a special time or over a short period, and do not capture the dynamics between stressful situations and individuals.

The Symptom Check List90 (SCL-90), Self-rating Anxiety Scale (SAS), and Self-rating Depression Scale (SDS), which are the most commonly used self-assessment scales, suffer from normative and non-invasive effects. The most commonly used self-assessment scales, such as SAS and SDS, have not been updated promptly, have been used inappropriately, resulting in contradictory results, lack a comprehensive understanding of the sample and scope of SCL-90 and other normative scales, and have problems of empirical validity for mental health measurement, which cannot accurately measure and evaluate mental health. However, the above scales do not target the special group of seafarers, and due to the problems of applicability of measurement objects and normative evaluation criteria^17^, they lead to a negative evaluation of seafarers' psychological problems; at the same time, the results are not comparable due to the different perspectives and assessment criteria of various instruments^18–22^. Incorrect use or overuse, reliance on scale ratings, or mechanical interpretation of results often leads to conclusions that do not correspond to reality^23,24^, so there is a need to optimize the items of the current scales. In addition, the application areas of the above tests are biased towards the quality of life and do not apply to specific maritime domains related to seafarers' mental health.

To collect empirical data, semi-structured interviews were conducted with 283 key stakeholders, including seafarers, seafarer agencies, maritime university students, etc., referring to the Symptom Check List90 (SCL-90), Self-rating Anxiety Scale (SAS), and Self-rating Depression Scale (SDS), and the Pilot Mental Health Scale (PMHS)^25^. Based on the scores of factors influencing the mental health of seafarers and interviews with maritime experts, and statistical analysis of the collected questionnaires.

### Fuzzy factor analysis of assessment factors

Since this paper used reference factors from the dimensions of the four scales, a total of 129 initial factors from the restructuring needed to be censored for irrelevant factors and analyzed for the restructuring factor control scale, represented by the naming of representative scale dimensions. Further analysis was therefore required for the factor scores obtained from the literature analysis and expert interviews.

The basic purpose of factor analysis is to use a few factors to describe the association between many factors, i.e., several scale variables with correlation are grouped in the same category, and each category becomes a factor (common factor), reflecting most of the information of the data with a few factors. The general factor analysis method is very sensitive to outliers, which can lead to crippling or erroneous analysis results. The relevant theory in fuzzy mathematics^26^ is introduced to improve the classical factor analysis and establish a fuzzy factor analysis model. The model makes up for the defect that classical factor analysis is vulnerable to the interference of outliers by fuzzy iterative calculation so that the influence of fuzzy information on the analysis results is reduced and the accuracy of the analysis results is improved^27^.

### Multi-factor correlation test

Since the listed factors are all positive indicators relative to the results, i.e., the higher the rating value of the factor, the stronger its effect on mental health^28^. For the results of factor scores from the literature analysis and expert interviews, the correlation matrix (Correlation Matrix) was calculated using SPSS v24 software. Some of the factors had a high correlation between them, reaching 0.9179. The Kaiser–Meyer–Olkin test statistic was 0.928 (KMO ≥ 0.900), and Bartlett's spherical test value was Sig. 0.01 (i.e., p < 0.05). It indicates that the variables are suitable for the factor analysis method, and the scale dimensions can be found by analyzing factors with correlations.

### Establishment of a fuzzy factor analysis model

A fuzzy factor analysis model for the factors of seafarers' mental health assessment can be established by introducing fuzzy factors.

1 $${X}_{m\times n}-F{E\left(F\right)}_{m\times 1}={L}_{m\times m}{F}_{m\times 1}+\varepsilon $$

where *F* is the dimension of the scale to be solved, *FE(F)* is the fuzzy mean coefficient, matrix *L* is the load matrix, and let *E*(ε) = 0.

2 $$FE(F) = \frac{{\sum\nolimits_{{\rm{k}} = 1}^{\rm{n}} {{{\rm{x}}_{\rm{k}}}{{\rm{U}}_{\rm{k}}}} }}{{\sum\nolimits_{{\rm{k}} = 1}^{\rm{n}} {{{\rm{U}}_{\rm{k}}}} }}$$

3 $$\text{F}=({L}^{T}L{)}^{-1}{L}^{T}(X-FE(F)$$

The scores of each factor can be obtained by the inverse of the matrix Where the fuzzy covariance and its corresponding eigenvalues ${\uplambda }_{x}$ and eigenvalue $\overrightarrow{{\text{e}}_{x}}$ are obtained.

4 $$\text{FCov}\left(\text{X},\text{F}\right)={\text{LL}}^{-1}$$

5 $$\text{L}=(\sqrt{{\uplambda }_{1}}\overrightarrow{{\text{e}}_{1}},\sqrt{{\uplambda }_{2}}\overrightarrow{{\text{e}}_{2}},\ldots,\sqrt{{\uplambda }_{x}}\overrightarrow{{\text{e}}_{x}},\ldots,\sqrt{{\uplambda }_{m}}\overrightarrow{{\text{e}}_{m}})$$

**Figure 1:**
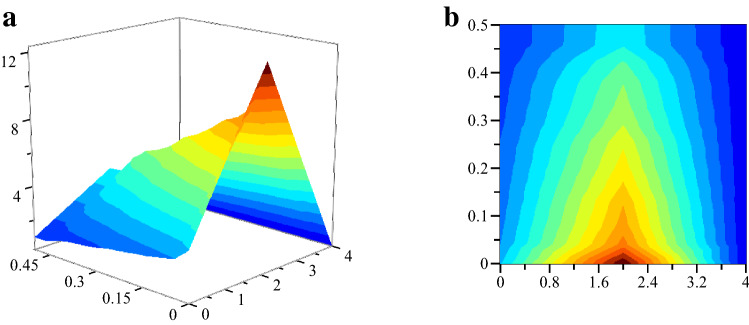
The three-dimensional relationship between the eigenroots, variance contribution rate, and correlation coefficient ratio is shown in the figure. (**a**) Three-dimensional relationship diagram of the parameters of the fuzzy factor model. (**b**) The z-axis projection of the three-dimensional relationship diagram.

The estimation method of fuzzy factor loading matrix in fuzzy factor analysis model is the same as the classical factor analysis model method. The fuzzy factor model is established, and the fuzzy eigenvalues, eigenroots, eigenvectors, fuzzy factor loadings, cumulative variance contribution, and fuzzy factor scores are calculated^29^. The fuzzy mean and fuzzy deviation were performed for 30 and 50 iterations, respectively, and the accuracy of the iterations was controlled within one ten-thousandth. The fuzzy covariance matrix $\text{FCov}{(X,F)}_{N\times N}$ of the seafarers' mental health assessment factors with eigenroots greater than 1 was calculated using MATLAB software (Fig. 1a,b).

Three dimensions were added to the usual scale dimensions, including "sexual cognitive abnormalities", "family influence", and "negative attitude toward emptiness". Due to the overlap in dimension assignment, the important hidden dimension of low self-esteem was no longer included in separate dimensions but was incorporated into "self-perception bias", which reduced the information crossover between indicators and established a preliminary macroscopic detection dimension. The final list of dimensions was determined by using the Likret 5-point scoring method to score the frequency of the dimension factors (g = 1, 2, 3, 4, 5)^30^.

According to the feature root results, thirteen scale dimensions were obtained in this paper, and the final total factor loadings contributed 90.18%, so these 13 dimensions were selected as the scale dimensions of the mental health scale, and the dimension naming needed to contain most of the information of the original factors and exist as a common factor. In this paper, we refer to the naming rules of four scales SCL-90, SAS, SDS, etc., and summarize the naming with higher semantic clarity, as shown in Table 1.

**Table 1:** Weighted ranking of subjective weights of survey dimensions.

Survey dimension	Total number of votes	Dimensional weighting	Weight ranking
Self-perception bias C8	259	932	1
Depression cognitive symptoms C4	271	921	2
Anxiety psychiatric symptoms C5	250	897	3
Family influence C12	244	881	4
Social impairment C3	201	728	5
Empty negative attitudes C13	232	698	6
Living environment experience C10	178	526	7
Physical abnormal symptoms C1	142	459	8
External environmental terrorC7	102	312	9
Conscious hostilityC6	96	256	10
Sexual cognitive abnormality C11	109	249	11
Obsessive–compulsive symptoms C2	86	159	12
Psychotic impulses C9	76	82	13
Pirate threat C14	n < 142 *	–	–
Temperature change C15	–	m < 80 *	–

^1^n = 283. This statistic is based on relatively subjective results of respondents for the mental health measure dimensions, with a small bias in the ranking, and the table only shows the main influences that met the statistical criteria. When n < 142 and m < 80.

### Item analysis

COSMIN (Consensus-based Standards for the Selection of Health Measurement Instruments) is a valid and reliable tool for evaluating the quality of health measures, and its purpose is to provide a clear framework for assessing the psychometric properties of the measure^31^. COSMIN uses a nine-domain quality rating system that includes internal consistency, reliability, measurement error, content validity, structural validity, hypothesis testing, cross-cultural validity, scale validity, and responsiveness.

For the obtained seafarers' mental health scale (SMHT), see the supplementary file for details, with the help of IBM SPSS Statistics v24 statistical analysis software, the scale was analyzed to find out whether it meets the mental health requirements. Firstly, the dimensions and factors corresponding to the initial scale were partially deleted, modified, added, incorporated, etc. For example, the modified items: (feeling that most colleagues on board are not trustworthy, fearing to participate in some collective activities on board, often feeling poor sleep quality at sea, fearing empty deck space, feeling that everything needs to be done quickly).

### Discriminant analysis method

Discriminant was calculated according to the critical ratio method^32^, and the total scale scores of each predictor were ranked high and low, and the items with no statistically significant difference (p > 0.05) and overlapping items were deleted, and "have some thoughts that others do not have", "miss my family ", "distressed by some thoughts about "sex", "strange and scary scenes in the brain", "screaming or dropping things", and "having a bad mood", "yelling or dropping things", etc.

### Frequency analysis

The frequency of the scores on the SMHT scale was found to deviate from the normal distribution for the frequency of "fear of being attacked by pirates while on a sailing mission", probably because this rarely happened in the sample, so it was replaced by "feel afraid ".

### Reliability analysis

Using Cronbach's alpha (Cronbach's alpha or Cronbach's α) total coefficient results show that 0.852 (0.80–0.90), with a good level of internal consistency reliability; retest reliability value (R-value) is 0.873 (0.85–0.90) using the formula (6). Reflecting the stability and consistency of the test across time.

6 $$\alpha =\frac{K}{K-1}\left(1-\frac{\sum_{i=1}^{K} {\sigma }_{{Y}_{i}}^{2}}{{\sigma }_{X}^{2}}\right)$$

### Validity of the validity scale correlations

Against the standard SCL-90, it can be concluded that (${\text{r}}_{i}$ = 0.468 ~ 0.841) > 0.45 using the formula (7). It reflects the better degree of validity of the behavioral performance of the SMHT scale test, which can correctly assess the mental health of seafarers.

7 $${r}_{i}=\frac{\sum (X-\overline{X })(Y-\overline{Y })}{N{S}_{X}{S}_{Y}}$$

### Scale readability

The readability rating of the SMHT scale was 7.6 using the Flesch-Kincaid formula (8). The results indicated that the readability of the scale met the basic requirements.

8 $$L=206.835-1.015\left(\frac{{T}_{w} \, }{{\text{T}}_{se}}\right)-84.6\left(\frac{ \, {T}_{sy} \, }{ \, {T}_{w}}\right)$$

Seafarers are faced with increasing job stress in terms of professional competition, technological updating, safety responsibilities, interpersonal relationships, and teamwork, which may have adverse effects on seafarers' physiology, psychology, and behavior, and thus produce a large proportion of human factors in maritime accidents^33^. If a mental health scale is simply used, subjectively biased behaviors may be present, ignoring the phonetically and semantically true emotional expressions of the person being tested. In this paper, we use a dual subjective and objective mental health testing method to reflect the true situation of mental health more comprehensively.

### A subjective mental health detection module

The character traits of seafarers are a complex sociological concept with more influencing factors, so they cannot rely too much on scale detection, and the traditional mental health assessment is mainly based on self-assessment questionnaires and structured interviews^34^, which are used to obtain data information from the subjects through face to face interpersonal interaction mode and to assess the mental health status of the subjects. Traditional mental health assessment methods objectively obtain the mental health status of the subjects from the perspective of scales, but there are problems of biased assessment responses^35^, poor real time assessment^36^, and passive assessment in the implementation process and assessment work^37^.

To this end, we mainly rely on deep learning methods based on natural language processing to obtain the subjective cognitive evaluation of seafarers' influence on a factor for a similar expert interview with seafarers and adjust the detection and evaluation mechanism of the traditional seafarers' mental health scale to make the results more consistent with the real seafarers' mental health evaluation.

The current ability to measure MHL has significant limitations, all of which are incomplete, and there is significant room for the development and evaluation of psychometrically robust measures to assess MHL-related attributes^38^. The assessment of mental health needs to focus on the adequacy of the current definition of MHL on the more subjective and objective perspective should be analyzed comprehensively, this paper uses a revised scale and a natural language processing-based approach to accomplish the purpose of the study mental health detection.

The analysis of the mental health assessment instruments was based on the semantic content of the questionnaires and interviews, rather than on the patients' responses to these questions^39^. Thus, although the final symptom categories used here are as semantically different as possible, they may not be independent of the underlying etiological perspective, just as high fever and fatigue are semantically different but may derive from the same underlying cause.

### Subjective speech emotion detection

In the 1970s, Albert Mehrabian, a professor of psychology at UCLA, pointed out in his articles "Decoding of Inconsistent Communications" and "Inference of Attitudes from Nonverbal Communication in Two Channels" that when people communicate verbally: 55% of the information is conveyed visually, such as gestures, expressions, appearance, makeup, body language, gestures, etc.; 38% of the information is conveyed auditorily, such as the tone of speech, voice intonation, etc.; and only 7% comes from pure verbal expression^40^.

**Figure 2:**
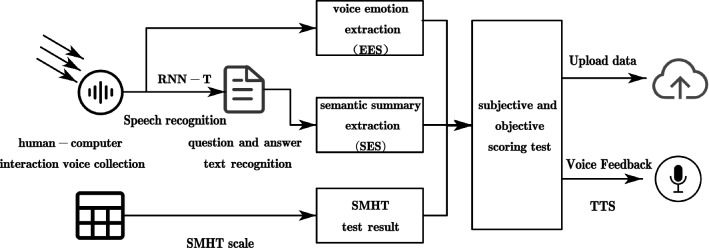
Schematic diagram of the dual subjective and objective mental health testing system.

The current psychological testing methods mainly focus on group or individual mental health scale tests and expert interviews, which greatly neglect the real emotional expressions shown by voice intonation and vocal tone during the testing process. In addition, traditional mental health tests are more inclined to mental health scale tests, which generally have certain subjective bias and cannot well represent the real mental health condition. Therefore, in this paper, the revised SMHT scale is used as the objective detection part of the auxiliary detection, and the subjective detection part of mental health is expressed based on feature comparison such as semantic extraction of text content summary and speech emotion extraction in speech communication by deep learning. Through the scoring of the above subjective and objective questions, the true mental health state of the subjects can be measured better and more accurately in the end, As shown in Fig. 2.

### Speech emotional extraction (SEE) model

**Figure 3:**
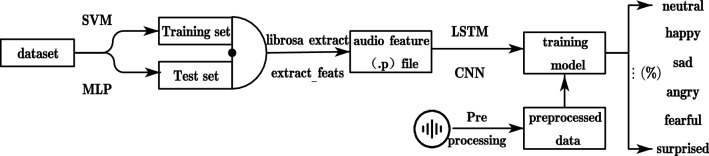
Speech emotion extraction model (SEE).

This section focuses on the extraction of emotions implied by the speech itself, by extracting the features of speech focusing on the characteristics of speech such as intonation, frequency, pitch, etc. For using the neural network structure of LSTM, CNN1, CNN2, the speech corpus of CASIA is used, by classifying the training set and test set and performing data cleaning and other pre-processing work, to represent the degree of the emotion of speech, after the database extraction, this paper sets a total of 6 different emotions: neutral, happy, sad, angry, fearful, and surprised. LSTM, as a special RNN structure, can accomplish the task in a better text list, which can solve the problem of gradient disappearance and gradient explosion due to long sequences in the training process^41^, and will have better performance, The full name of LSTM is Long Short Term Memory, which is a neural network with the ability to remember long and short term information. LSTM is composed of a series of chained LSTM units (LSTM Units), and the gate mechanism is introduced to control the circulation and loss of features, with the purpose of solving the long-term dependency problem. Convolutional Neural Networks (CNNs) are a class of Feedforward Neural Networks (FNs) that include convolutional computation and have a deep structure, including a series of convolutional, pooling, and fully connected layers. The purpose is to be able to perform shift-invariant classification of the input information according to its hierarchical structure. Figure 3 demonstrates the speech emotion extraction model.

**Figure 4:**
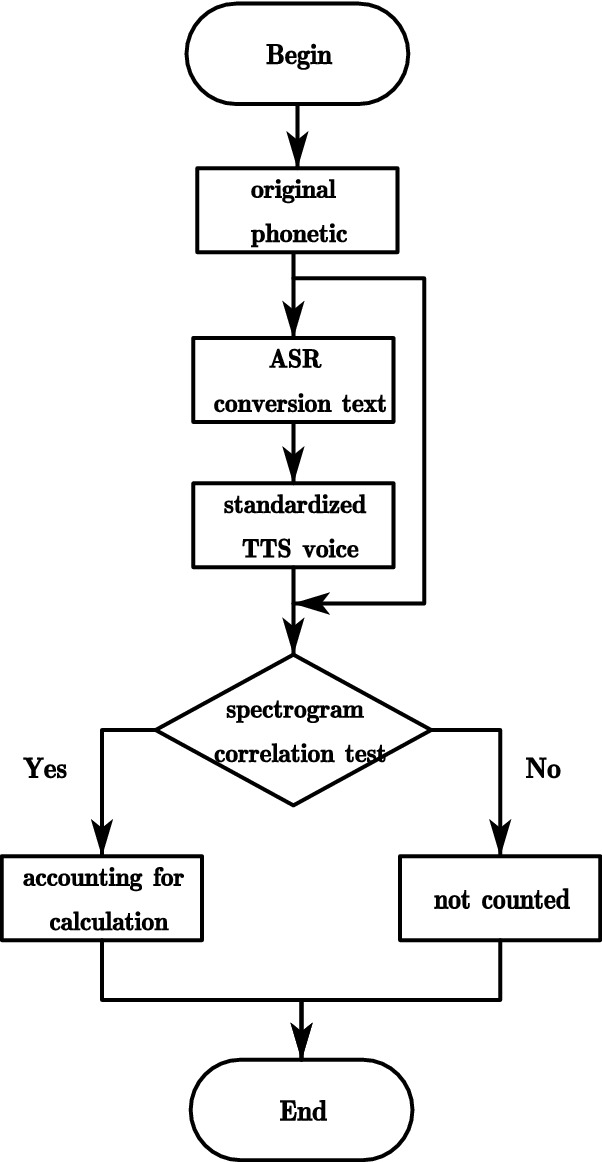
Schematic diagram of TTS matching mechanism.

The classification of the data needs to be performed first, and then the trained model is tested to get the final classification results. Among them, in conducting the correlation problem on the intonation of speech, TTS matching is used to check the overlap of waveform graphs, and Fig. 4 shows the flow chart of TTS matching mechanism.

### Semantic summary extraction model (SSE)

The implementation of semantic summary is the process of obtaining the core ideas, summaries, and keywords of text information through automatic computer recognition of natural language processing when the subject answers relevant questions in the process of human–computer interaction. The significance of this processing is that it can solve the problem of subjective bias in the objective question response module (mental health scale), directly extract the key information of the subject's question responses, and provide an important method for further. It provides an important identification method to match emotions or feelings.

**Figure 5:**
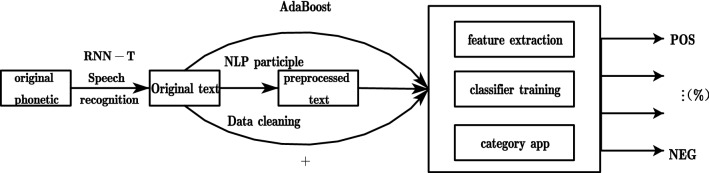
Schematic diagram of SSE workflow.

Sentiment Analysis, also known as Opinion Mining, is the process of analyzing, processing, summarizing, and then reasoning about subjective text with emotional content^42^. The extraction process is mainly through speech recognition based on RNN-T, which can recognize speech-to-text information with high accuracy online in real time. The preliminary complete text is obtained, and then, the acquired text is preprocessed and the sentiment analysis of the preprocessed text is performed using plain Bayes to obtain the degree percentage of a certain level of negative, objective, and positive for the segments of this paper. AdaBoost is an iterative algorithm that trains different classifiers for the same training set, and then aggregates these weak classifiers to form a stronger final classifier. Finally, AdaBoost is used again: a weighted majority voting method is adopted to increase the weights of those samples that were incorrectly classified by the previous round of weak classifiers and decrease the weights of those samples that were correctly classified to classify the whole classification results for classification strengthening, and the schematic diagram of SSE work is shown in Fig. 5.

**Figure 6:**
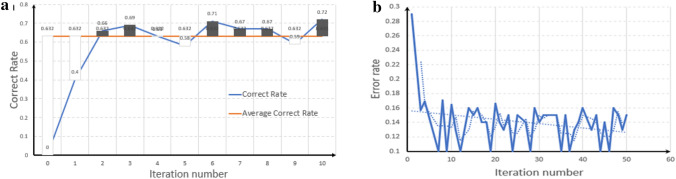
Schematic diagram of the accuracy rate of the two methods. (**a**) Accuracy of plain Bayesian. To ensure the efficiency of the method, attention is paid to the performance effect during the initial period, when the effect curve shows a good climbing ratio of 33 n/C_r_ (n → Smaller) with only the initial number of iterations, when the threshold of the mean correct rate is exceeded and increases exponentially as the program progresses. (**b**) Schematic diagram of binary classification accuracy. Recognition efficiency is greatly improved when the program acquires steep change curves as it carries out the learning process and fluctuates to reduce the recognition error rate to within a reasonable upper and lower threshold interval in accordance with fitting the expected mean-expectation trajectory.

From the (Fig. 6a,b) we can see that the AdaBoost dichotomous error rate and polynomial plain Bayes performed better, the model's recognition correct rate gradually becomes larger with the increase in the number of iterations, and the further the correct rate is much greater than the average correct rate, the correction module consisting of AdaBoost, with the increase in the number of times greatly reduces the error rate, the correct rate remains between 81 and 84%, and has a better classification effect on the semantic expression of the text.

The final scoring criteria indicated that since the objective assessment is mainly a modified seafarers' mental health scale, which has a good form of emotional expression to a large extent, the ratio of subjective assessment was appropriately reduced to 6:4 according to the current SSE and SEE methods, which have fewer data. According to the "7-38-55" rule of Professor Albert McLabin, the ratio of objective assessment value, text semantic summary value, and speech emotion extraction value is defined as 6:1.5:3.5 in this paper. The collected test speech was model tested and face to face interviews were conducted with 10 test subjects, and the comparison results revealed that the scores against the seafarers' mental health status decreased in 4 and increased in 6. Among the 8 members who met the interview results, the accuracy of the SMHT based dual subject-objective detection system increased by 12.05% relative to the scale detection alone, which greatly improved the accuracy of mental health detection, which provides a better auxiliary solution for further research on the mental health status of seafarers.

### Comparison with current methods/discussion

In this paper, we firstly improved the current commonly used mental health scale by adjusting the items and applying fuzzy factor analysis to obtain the scale dimensions of mental health assessment as 13 dimensions, and renamed the dimensions to make it more representative of many assessment factors, and conducted reliability and validity tests by SPSS software, and the results showed that SMHT can meet the requirements of measuring seafarers' mental health. The results showed that SMHT can meet the requirements of measuring seafarers' mental health. The subjective and objective tests were used, based on the improved mental health scale SMHT as an important basis for objective scoring. The comparison of features such as summary semantic extraction (SSE) of text content and emotion extraction (SEE) of speech intonation based on deep learning in speech communication was used to represent the subjective part of mental health. By scoring the above subjective and objective questions, the true mental health status of the subject can eventually be measured more accurately.

Gibbons et al. examine the computerized adaptive diagnosis and testing of mental health disorders, exploring recent advances in computerized adaptive diagnostic screening and computerized adaptive testing for the presence and se machine of mental health disorders (e.g., depression, anxiety, and mania); Arkaprabha Sau. et al. examine the use of machine learning techniques to screen seafarers for anxiety and depressive mood, exploring text-based effective calculations, and constructing mental health assessment models.LR Derogatis compile the SCL-90 Mental Health Scale for seafarer mental health research using traditional research by questionnaire data statistics; Shi, Y. study the comparative analysis of the fMRI low frequency amplitude ratio in seafarer psychological assessment.

Table 2 compares the advantages and disadvantages of SMHT, CAT, multimodal, SCL-90, and fMRI techniques, respectively. In this paper, the emotion expression implied by speech itself is investigated by scale detection, text semantic summary extraction (SES), and emotion extraction of speech intonation (EES) using a dual subject-objective detection approach, which makes the present method more advantageous relative to other research tools for assessing the ground.

**Table 2:** Comparison of the advantages and disadvantages of SMHT with the representative four methods.

Authors	Type of test	Disadvantages	Advantages
This paper	SMHT	Small sample size	More comprehensive factor analysis in the testing scheme, focus on dual subject-objective mental health testing, greatly improves scale modification, captures hidden emotional expressions
Gibbons	CAT	The narrower range of applications	High flexibility
Arkaprabha Sau	machine learning classifier	Model instability, local features	Simple, easy
LR Derogatis	SCL-90	Not suitable for maritime domain	Classical test theory
Shi, Y	fMRI	Not suitable for daily mental health monitoring in the maritime domain	Physical tests, focus on subjects' low frequency amplitude ratios, provide quantitative early warning models

## Results

This paper adopts a dual subjective and objective mental health testing composition scheme. Firstly, combining the results of literature analysis and seafarer interview survey, the fuzzy factor analysis method is applied to revise the new Seafarer Mental Health Test Scale (SMHT). Then we collected test data from 283 marine related practitioners and the SPSS evaluation results of the scale showed good reliability (total Cronbach's alpha value of 0.852 $\in$(0.80 ~ 0.90), retest reliability R of 0.873 $\in$(0.85 ~ 0.90)) and scale association validity (for the standard SCL-90 scale, ($\text{r}$ = 0.468 ~ 0.841) > 0.45). The results indicate that the scale meets the requirements of mental health testing. An immersive subjective emotion extraction is based on natural language processing, i.e. semantic summary extraction (SSE), speech emotion extraction (SEE). Finally, we took the collected detection data into a mixed scoring mechanism and obtained the final scores. The results improved the correction degree by about 12.05% compared with the traditional mental health scale, which showed a more accurate psychological detection effect. In addition, the method was compared with CAT, Machine Learning, SCL-90, fMRI, and other mental health testing techniques, and the dual subject-objective testing technique has more prominent testing advantages. The method can provide a better auxiliary solution for standardized seafarer psychological testing.

The performance of the trained model was tested by randomly selecting 40 of the 283 survey participants who had completed the SMHT scale test, and 29 valid responses were received, with a recovery rate of 72.5%, thus initially satisfying the requirement for a valid questionnaire. As shown in Table 3.

**Table 3:** Performance measurement table for the dual detection model in the paper.

RNN-T (WER, %)	Self-test error rate (self-TER, %)	Acc	The calibration rate
7.81	10.73	0.89	12.05%

RNN-T (WER, %) refers to the word error rate of end-to-end speech recognition speech-to-text conversion of RNN-T; self-test match (self-cm) refers to the difference quotient of scores after the dual subject-objective test and the seafarer mental health test SMHT only. The self-test process is somewhat personal and subjective, so the data may have slight bias.

## Discussion and conclusions

The paper focuses on the detection method of seafarers' mental health detection, which extracts and detects the emotions of speech (SEE) and text (SSE) through a dual subjective and objective detection module based on natural language processing. It effectively avoids the subjective bias of the traditional seafarers' mental health detection scale and pays more attention to the hidden emotional expressions of speech itself. The improved seafarer's mental health scale SMHT is based on the existing SPSS v24 software for in-depth validation and revision of the entries to further improve its applicability in the restricted range of seafarer's group in the seafaring range. It can be used as a tool to assess the mental health of seafarers.

Mental health testing of seafarers is an important step in ship and sea management that requires continuous improvement. We have not completely abandoned the role of mental health scales in seafarers' mental health testing, but on the contrary, we have given full recognition to the mental health scales that have been used for a long time in medical, industrial, and educational industries for patients, employees, and students, and have regained the exclusive information on the mental health factors affecting seafarers in this particular profession through literature analysis and semi-structured interviews. The newly created Seafarers' Mental Health Test Scale (SMHT) has a good detection effect by reacquiring the exclusive detection dimensions (13 dimensions) through literature analysis method and semi-structured interview method and conducting sufficient research and data feedback on each detection dimension to obtain 6–10 different numbers of detection factors (121 in total). At the same time, the preliminary scale was tested for reliability and validity, and the scale was checked comprehensively by overlapping deletion, missing addition, and errata modification to obtain a standardized scale that could satisfy the seafarers' mental health testing, and the scores obtained from the scale were used as the result composition of the objective testing library module through the established testing framework of voice and text interaction.

The scale alone is limited to a large extent, ignoring the real emotions of the interviewees in the testing process. The scale is more objective in its presentation and the scoring criteria are superimposed and therefore more homogeneous. The professional expert interview has better mental health test results, but it is not suitable for use as a daily psychological test of seafarers. In this paper, we use the objective test module of mental health scale, and at the same time, we use the deep learning-based emotion detection method to detect the emotion (SSE, SEE) in the speech and text parts of the interviewees' responses in the subjective question bank, and based on the "7-38-55" rule of Professor Albert McLabin, we initially obtain the objective and subjective psychological test based on the SMHT scale. The results of the SMHT scale, which is a subjective and objective mental health testing system, showed good experimental results. We still believe that this method of testing will largely improve the authenticity of the seafarer mental health test, but at the same time, we are constantly studying how to obtain more refined scoring criteria for both subjective and objective tests, so as to distinguish the effects of small changes in scoring at different levels.

In addition to the paperless human–computer interaction test, research has shown that a significant number of respondents to the mental health test were not satisfied with the results of the mental health scale because they expressed internal resistance to the initial known purpose of the test, which influenced the selection of the paper-based test. A large part of the reason we adopted a paperless approach was to reduce the resistance of respondents to mental health testing, and the use of voice interaction (TTS, ASR) not only greatly improved the efficiency of the test, but also the authenticity of the test. This system can upload data to the cloud for mental health surveillance and visual monitoring of group seafarers' mental will health monitoring, which provides scientific data support for further proposed solutions.

## Prospects

The accuracy and efficiency of the natural language processing-based seafarer mental health detection model depend heavily on the sample size of natural language. The deep learning architecture in image processing can also be used to detect mental health problems from facial or body language. The next step of this study is to investigate the comparison of the accuracy rate change caused by increasing the sample size of natural language, and multimodal emotion recognition detection of micro-expressions based on vision modules.

## Supplementary Information

Supplementary Information.
