# Supplementary material for: A deep learning based method for intelligent detection of seafarers' mental health condition
Source: Sci Rep. 2022 May 12;12:7890. doi: 10.1038/s41598-022-11207-7 (PMC9098153; doi:10.1038/s41598-022-11207-7)
Supplement: Supplementary file 1 — Supplementary Information. [file 41598_2022_11207_MOESM1_ESM.pdf]

Appendix A

Table A. Details of mental health testing factors for seafarers

Serial number	Measurement factors	Serial number	Measurement factors
1	Felt that most colleagues on board were not to be trusted8	62	Feeling unnatural with the opposite sex11
2	Never felt good about relationships with others9	63	Decreased sexual desire11
3	Have unwanted thoughts or phrases swirling around in my head2	64	Poor appetite10
4	Have thoughts that no one else has8	65	Constantly concerned about what others think of you8
5	Feeling scared or fearful for no reason5	66	Feeling that others do not understand you and do not empathize with you3
6	Feel fidgety and distracted5	67	Fear of fainting in public7
7	Feeling guilty10	68	Can't sleep10
8	Feeling that your colleagues on board are privy to your private thoughts9	69	Feeling that someone is watching you or talking about you8
9	Blaming yourself a lot4	70	Disinterested in things4
10	Shaking all over5	71	Waking up too early10
11	Feeling isolated by your colleagues on board13	72	Masturbation11
12	Back pain1	73	Frequent sexual fantasies11
13	Unnecessarily double-checking current things at work2	74	Feeling difficulty in completing tasks given by superiors2
14	Feeling painful4	75	Worrying too much4
15	Your mood is extremely vulnerable3	76	Not initiating phone calls or online chats12
16	Fear of participating in some group sexual activities on board7	77	Feeling tingling or tingling in the body1
17	Feeling shy and uncomfortable when being with the opposite sex3	78	Don't know how to organize free time13
18	Troubled by thoughts about sex9	79	Feeling that I have little value4
19	Numb yourself by smoking, drinking alcohol or coffee13	80	Being a failure myself8
20	Not having many expectations of yourself8	81	Life at sea has no purpose13
21	Afraid to perform tasks alone7	82	Difficult to make a choice2
22	Missing family12	83	A bout of chills or fever1
23	Life is dull and boring13	84	Headaches1

24	Thinking about death10	85	Having violent urges to hit or hurt others6
25	Inefficient in doing things2	86	Feeling uncomfortable eating in public3
26	Feeling nervous and anxious when alone7	87	Perceived low weight of family in career plans12
27	Feeling lonely even with others9	88	Dizziness or fainting1
28	Can't work properly because I miss my family12	89	Often worry about being well-dressed and properly groomed2
29	Feeling uncomfortable in a crowded place7	90	Heartbeats very hard and abnormally5
30	Nausea or stomach discomfort1	91	Fear of empty deck space7
31	Nervousness and insecurity5	92	Feeling that everything is difficult4
32	Feeling that something familiar is suddenly strange5	93	Yelling or dropping things6
33	Feeling that others are trying to take advantage of you8	94	Feeling that there is something seriously wrong with your body9
34	Frequent desire to argue with others6	95	The perception that others are not making appropriate comments about your accomplishments8
35	Trouble because sexual needs are not satisfied for a long time11	96	Low motivation to eat10
36	Having to wash your hands repeatedly and feeling dirty2	97	Decreased interest in the opposite sex4
37	Avoiding certain things, situations, or activities because you feel afraid7	98	Have difficulty breathing1
38	Feeling tense or easily nervous5	99	Hearing voices that cannot be heard by others9
39	You think you should be punished for your faults9	100	Feeling paranoid about others3
40	Chest pain1	101	Feeling hopeless about the future4
41	Feeling heavy and weak in your hands or feet1	102	Sleeping in a trance10
42	Feeling inferior to others in ability3	103	Hate yourself8
43	Distressed by thoughts about "sex "11	104	Muscle aches1
44	Can't control losing your temper6	105	Difficulty falling asleep10
45	Indulged in abnormal sexual behavior11	106	Feeling weakness in a part of the body1

46	Often blaming others for causing problems8	107	No confidence in your ability8
47	Feeling inferior to colleagues in many ways8	108	Can't concentrate2
48	Ashamed of exploring the seafaring profession8	109	Thinking your family doesn't like you12
49	Nothing to do13	110	Feeling that your energy has decreased and your activities have slowed down4
50	Throat obstruction1	111	Mind becomes blank2
51	Strange and scary scenes9	112	Don't feel happy at home12
52	Easily annoyed and agitated6	113	Feeling that everything needs to be done quickly5
53	Feeling lonely4	114	Unconditionally tolerate reproaches from others3
54	Feeling scared5	115	Sexual matters prevent me from concentrating on my work11
55	Feeling uncomfortable when others look at you or talk about you3	116	Feeling that others can control your thoughts9
56	Often eat irregularly10	117	Want to end my life4
57	A bout of fear or panic5	118	Crying easily4
58	Frequent forgetfulness of memories2	119	Have thoughts of breaking or destroying things6
59	Feeling cheated, trapped, or that someone is trying to catch you4	120	Feel like something is wrong with your mind9
60	Often feel self-conscious about the poor quality of sleep at sea10	121	Have nightmares10
61	Feeling that people are not friendly to you and do not like you3		
